# A Two-Stage Prediction Strategy for Primary Hyperparathyroidism

**DOI:** 10.3390/diagnostics16193200

**Published:** 2026-10-02

**Authors:** Ruisi Chen, Min Ren, Jingyan Chen, Pin Wang

**Affiliations:** 1 Department of Endocrinology, School of Medicine, University of Electronic Science and Technology of China, Sichuan Provincial People’s Hospital, Chengdu 610072, China; ruisi9295@163.com; 2 Department of Endocrinology, Sichuan Provincial People’s Hospital, School of Medicine, University of Electronic Science and Technology of China, Chengdu 610072, China; minminshiyumi@126.com

**Keywords:** primary hyperparathyroidism, parathyroid hormone, risk prediction model, logistic regression, external validation

## Abstract

**Background:** Primary hyperparathyroidism parathyroid hormone (PTH) hypersecretion is difficult to recognise when calcium is normal and difficult to distinguish from secondary hyperparathyroidism once PTH is found to be elevated. These are separate problems arising at separate points in care. We developed and evaluated two interpretable models corresponding to these decision points using an internal–external validation design. **Methods:** We analysed 2788 adults undergoing PTH testing across three campuses of one hospital (development *n* = 1843; internal–external validation *n* = 445 and *n* = 500); those with an estimated glomerular filtration rate below 30 mL/min/1.73 m^2^ were excluded. PHPT was adjudicated in 504 (18.1%) by two endocrinologists using biochemistry, imaging and histopathology. Model 1, for use before PTH is measured, comprised total calcium, ln(alkaline phosphatase) and age. Model 2, developed in the Main campus subgroup with PTH above the reference limit (>65 pg/mL, *n* = 429 of 623), comprised ln(PTH), total calcium and phosphate. **Results:** Model 1 achieved AUCs of 0.920 in the development cohort and 0.882–0.908 in the validation cohorts; because serum calcium contributed to the outcome definition, these estimates reflect agreement with the reference standard rather than independent diagnostic accuracy. Among participants with elevated PTH, Model 2 achieved an AUC of 0.904 in the development cohort and 0.892 in the pooled validation cohort compared with 0.803 and 0.791 for PTH alone, respectively. In the normocalcaemic subgroup, Model 2 achieved an AUC of 0.790 compared with 0.699 for PTH alone. Applied sequentially, the models referred 18.0–26.1% of participants while capturing 78.1–85.0% of PHPT cases and identified a second-stage subgroup with an observed PHPT prevalence of 93.1–95.1%. **Conclusions:** This two-stage strategy may support further testing and referral for PHPT using routinely available measurements. Prospective validation in broader clinical populations is required before clinical implementation.

## 1. Introduction

Primary hyperparathyroidism (PHPT) arises when PTH secretion becomes uncoupled from calcium–phosphate feedback regulation [1,2,3]. Sustained PTH excess contributes to skeletal, renal and metabolic complications [4,5,6]. As contemporary PHPT is increasingly mild and biochemically subtle, two distinct diagnostic problems have become prominent: recognising PHPT when calcium is normal and distinguishing autonomous PTH secretion from secondary hyperparathyroidism once PTH is elevated [7,8,9,10,11].

The first problem concerns testing. Suspicion is commonly triggered by an elevated serum calcium, and PTH, a central test in the diagnostic evaluation, may not be measured when calcium is normal [12]. In our cohort, 113 of 504 PHPT cases (22.4%) had total calcium at or below the local upper reference limit, and a total-calcium threshold alone would therefore have identified 77.6% of cases. At this stage, a practical method is needed to identify patients who should undergo PTH testing.

The second problem concerns attribution after PTH elevation has been identified. Current guidance recommends excluding secondary causes using ionised calcium, 24 h urinary calcium, repeated PTH measurement and reassessment after vitamin D repletion [7,8]. These investigations are not uniformly available outside specialist centres. Among participants with elevated PTH and normal calcium in our cohort, 61.2% were ultimately classified as not having PHPT. Several biochemical indices have been proposed to address this problem using routinely available analytes [13,14,15,16], but their ability to distinguish normocalcaemic primary hyperparathyroidism from vitamin-D-related secondary hyperparathyroidism remains uncertain [17]. This limitation is also relevant to the predominantly vitamin-D-deficient comparison group in the present study. We therefore approached PHPT prediction as two related questions at different points in care: whether PTH should be measured and, once elevated, whether the elevation reflects autonomous secretion or a secondary cause. We hypothesised that the calcium phosphate phenotype, reflecting the renal phosphaturic effects of PTH, would provide information beyond the PTH concentration alone. In this multicentre retrospective study across three campuses of Sichuan Provincial People’s Hospital, we developed two interpretable models corresponding to these decision points and evaluated them using an internal–external validation design. We assessed discrimination, calibration and clinical utility and compared the second model with previously published biochemical indices applied without recalibration. Reporting follows the TRIPOD+AI statement [18]. Both models are intended to support further testing and referral rather than to independently establish the diagnosis.

## 2. Materials and Methods

### 2.1. Study Design and Participants

#### 2.1.1. Study Population

We conducted a multicentre retrospective cohort study across three campuses (Main, East and Caotang) of Sichuan Provincial People’s Hospital. Adults aged 18 years or older who underwent serum PTH testing between September 2018 and October 2024 were consecutively identified from the electronic medical record system (*n* = 5769).

Participants were excluded if they were pregnant or lactating (*n* = 13); had a malignancy, severe acute or chronic infection, an established haematological disorder or severe liver disease (*n* = 315); had undergone previous parathyroidectomy (*n* = 98); had more than 20% of the 22 collected clinical variables missing (*n* = 875); had received calcimimetics, thiazide diuretics, glucocorticoids or lithium within the preceding three months (*n* = 277); or had received bone-forming or antiresorptive agents, including teriparatide and bisphosphonates, within the preceding year (*n* = 923). Participants with an estimated glomerular filtration rate (eGFR) below 30 mL/min/1.73 m^2^ (KDIGO stages G4–G5, *n* = 480) were also excluded because chronic-kidney-disease-related secondary hyperparathyroidism at this stage presents with an opposing mineral phenotype. These exclusions were applied because these conditions and treatments may alter calcium, PTH, alkaline phosphatase or bone metabolism and thereby compromise the reference standard.

**Figure 1:**
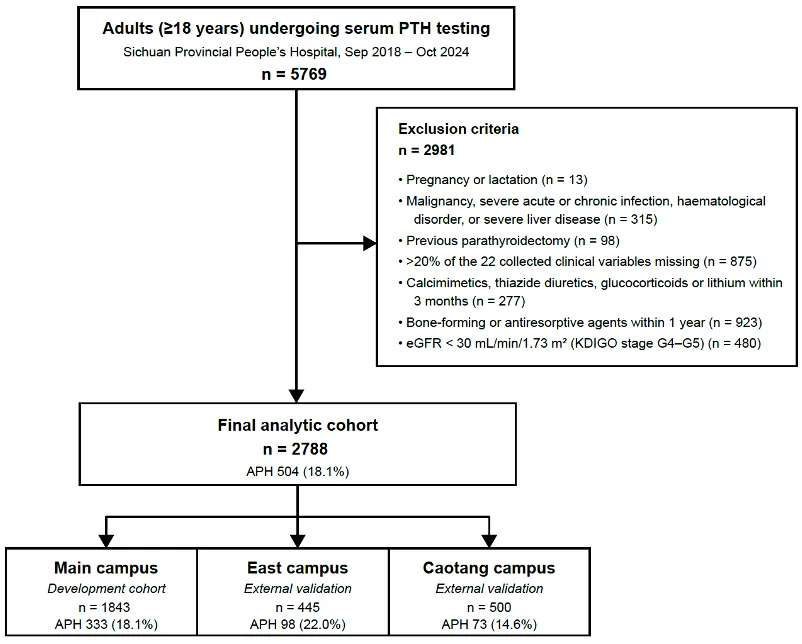
Flow of participants from adults undergoing serum PTH testing across the three campuses of Sichuan Provincial People’s Hospital to the final analysis cohort. Exclusions are shown grouped by the eligibility criteria applied at enrolment and by the additional exclusion applied for the present analysis. Participants with an estimated glomerular filtration rate below 30 mL/min/1.73 m^2^ (KDIGO stage G4–G5) were excluded because chronic-kidney-disease-related secondary hyperparathyroidism at this stage presents with an opposing mineral phenotype. The Main campus served as the development cohort, and the East and Caotang campuses served as external validation cohorts. APH, autonomous parathyroid hormone hypersecretion; eGFR, estimated glomerular filtration rate; KDIGO, Kidney Disease: Improving Global Outcomes; PTH, parathyroid hormone.

Of the 5769 adults screened, 2981 were excluded, leaving 2788 for analysis: 1843 at the Main campus, which served as the development cohort, and 445 at East and 500 at Caotang, which served as external validation cohorts (Figure 1).

This study was approved by the Ethics Committee of Sichuan Provincial People’s Hospital (Ethics Approval No. 752, 2024), with a waiver of informed consent for retrospective analysis of de-identified data.

#### 2.1.2. Reference Standard

All participants underwent PTH and calcium–phosphate testing at the index (baseline) visit. In accordance with routine outpatient practice at the participating campuses, participants whose PTH exceeded the local reference upper limit (65 pg/mL) in the presence of serum calcium within the local reference range (upper limit 2.52 mmol/L) received vitamin D repletion and were re-tested for PTH and serum calcium after approximately three months. Participants whose PTH normalised on re-testing were regarded as having vitamin-D-deficiency-related secondary hyperparathyroidism; those with persistently elevated PTH proceeded to ^99^mTc-MIBI SPECT/CT parathyroid localisation imaging (or ^18^F-fluorocholine PET/CT from November 2023) [19,20,21].

PHPT was defined as PTH above the local reference upper limit (15–65 pg/mL) confirmed on at least two occasions, together with either total calcium above the local reference upper limit (2.11–2.52 mmol/L) or calcium within the reference range and PTH persistently elevated after vitamin D repletion and re-testing. The diagnosis was supported, where available, by a focal parathyroid lesion on ^99^mTc-MIBI SPECT/CT or ^18^F-fluorocholine PET/CT interpreted alongside concurrent biochemistry or by histopathology from parathyroidectomy demonstrating a parathyroid adenoma, carcinoma, or hyperplasia consistent with autonomous function.

Secondary causes of PTH elevation were addressed before autonomy was inferred: vitamin D deficiency was corrected and PTH re-tested as described above; participants with an eGFR below 30 mL/min/1.73 m^2^ and those exposed to calcimimetics, thiazide diuretics, glucocorticoids or lithium within three months or to bone-forming or antiresorptive agents within one year were excluded at enrolment. Participants not meeting the above criteria were classified as non-PHPT; those whose PTH normalised after vitamin D repletion were regarded as having vitamin-D-deficiency-related secondary hyperparathyroidism and were included in this group. Participants with an eGFR of 30 to 60 mL/min/1.73 m^2^ were retained, since they form part of the outpatient population in which the models would be applied, whereas below 30 mL/min/1.73 m^2^ chronic-kidney-disease-related secondary hyperparathyroidism dominates the mineral phenotype. Twenty-four-hour urinary calcium, the calcium-to-creatinine clearance ratio, family history and CASR genotyping were not systematically available and therefore did not contribute to classification, so familial hypocalciuric hypercalcaemia could not be systematically excluded.

All classifications were made by two endocrinologists independently, with disagreements resolved by a third senior endocrinologist. Blinding of the adjudicators to the model predictors was not possible, since serum calcium and PTH form part of the reference standard itself. All predictor variables were obtained at the index visit; re-test, imaging and histopathology results contributed only to the reference standard. Localisation imaging was used to localise a lesion and was not by itself taken either to establish or to exclude the diagnosis. Where an imaging or histopathology result conflicted with the initial biochemical classification, the case was reviewed by two endocrinologists together with the biochemistry, the imaging and histopathology findings and any available follow-up data.

#### 2.1.3. Data Collection and Variable Definitions

Demographics, comorbidities, laboratory measurements (calcium–phosphate metabolism, bone turnover markers, renal function indices and other routine biochemistry), imaging results and histopathology were extracted from the electronic medical record system.

Serum calcium was measured by the NM-BAPTA colorimetric method, serum phosphate by the phosphomolybdate method and 25-hydroxyvitamin D by chemiluminescent microparticle immunoassay. Parathyroid hormone was measured by electrochemiluminescence immunoassay on a Roche cobas e 801 analyser (Roche Diagnostics, Singapore), a second-generation intact-PTH method, with a local reference interval of 15–65 pg/mL. Measured inter-assay coefficients of variation were 1.8% for calcium, 3.6% for phosphate and 8.7% for parathyroid hormone; analytical quality met the requirements of the national standard WS/T 403-2024 [22]. The inter-assay coefficient of variation for 25-hydroxyvitamin D was not available retrospectively.

### 2.2. Candidate Predictors and Model Specification

Two models were developed on clinical grounds to address two distinct decision points rather than to a nested sequence of predictors.

Model 1 addresses whether PTH should be measured. It comprised total serum calcium, natural-log-transformed alkaline phosphatase and age, selected on clinical grounds from variables available before PTH testing. The model was developed in all participants. Because every participant had undergone PTH testing, its performance was evaluated within a tested population; prospective evaluation would be required before it could be used to select patients for testing. Serum phosphate was not considered for Model 1 because it is not routinely available at the relevant decision point. At the participating campuses, total calcium is included in the routine electrolyte panel, whereas phosphate is generally measured only when a metabolic indication is already suspected. Although phosphate was nearly complete in the present cohort (2 missing values among 2788 participants), this reflects prior PTH-related evaluation and does not represent its availability in a general outpatient population.

Model 2 addresses the question that follows an elevated PTH. It was developed in the Main campus subgroup with PTH above the local upper reference limit (>65 pg/mL; 623 participants across all three cohorts, including 429 in the development cohort). The model comprised natural-log-transformed PTH, total serum calcium and serum phosphate. Because PTH contributed to both subgroup eligibility and outcome classification, performance estimates for Model 2 may be affected by incorporation bias. Within the PTH-elevated subgroup, however, the clinical question is attribution of PTH elevation rather than its detection.

Alkaline phosphatase and PTH were natural-log transformed because their distributions were markedly right-skewed. Total calcium was used because it was the most consistently available calcium measurement across the participating campuses and had been selected before model fitting.

### 2.3. Missing Data

Multiple imputation by chained equations (m = 10, Bayesian ridge regression) was the primary approach to missing data [23]. The imputation model included the outcome and 17 auxiliary variables and assumed that data were missing at random conditional on the included variables. Model coefficients and standard errors were combined using Rubin’s rules, and discrimination and calibration were computed from predicted probabilities averaged across imputations. Participants with more than 20% of the collected clinical variables missing were excluded before imputation because such records were considered likely to reflect a limited clinical work-up, in which imputed values would depend more on the imputation model than on observed data. This 20% cut-off was a pragmatic data-quality criterion rather than a validated statistical threshold. Within the retained cohort, missingness of the final predictors of both models did not exceed 0.2%. Missingness by variable and by outcome status is reported in Supplementary Table S3.

### 2.4. Model Development

Both models were fitted using unpenalised multivariable logistic regression with maximum likelihood estimation in the Main campus development cohort. Predictors were selected before model fitting based on clinical and pathophysiological considerations and their availability at the relevant decision point, rather than through an automated selection procedure. Because this selection was not documented in a prior protocol or statistical analysis plan, it was not considered formally prespecified.

Both models were finalised in the development cohort and subsequently applied to the East and Caotang validation cohorts using fixed coefficients and without refitting. After model finalisation, we conducted a post hoc exploratory analysis of all combinations of up to four candidate predictors available at each decision point. The validation cohorts were not used to select the final predictors. Serum phosphate was additionally included in the candidate pool for Model 1 to examine the potential performance cost of excluding a measurement that was not routinely available at the pre-PTH decision point. Details of the candidate-predictor analysis are provided in Supplementary Table S4.

The number of events per predictor was 111 for Model 1 and 37 for the minority outcome group in Model 2. Odds ratios are reported per one-standard-deviation increase in each predictor. Both models were fixed after development and applied unchanged in external validation. The complete model coefficients and unit conventions required to apply each model are provided in Table 1.

### 2.5. Internal Validation and Internal–External Validation

Optimism in the development cohort was estimated using bootstrap resampling with 200 replications. In each bootstrap sample, the model-fitting procedure was repeated, and performance was evaluated in both the bootstrap sample and the original development cohort to obtain optimism-corrected estimates [24]. The final models were then applied to the East and Caotang validation cohorts using fixed coefficients and without refitting. Discrimination was assessed using the area under the receiver operating characteristic curve (AUC), reported with DeLong 95% confidence intervals calculated using the DeLong method. Calibration was evaluated using calibration plots, calibration slope and calibration-in-the-large [25], and predictive performance was assessed using the Brier score.

### 2.6. Risk Stratification and Sequential Use

A three-tier risk-stratification scheme for Model 1 was defined in the development cohort and applied unchanged to the East and Caotang validation cohorts. The lower predicted-probability threshold (0.105) corresponded to 90% sensitivity in the development cohort, and the upper threshold (0.190) corresponded to 90% specificity. Participants were accordingly classified into low-, intermediate- and high-risk groups.

The two models were also evaluated as a sequential strategy. Model 1 was first used to identify participants for whom PTH and phosphate measurement would be recommended, after which, Model 2 was applied to those with PTH above the local upper reference limit of 65 pg/mL. Three Model 1 operating points were derived in the development cohort, corresponding to target sensitivities of 90%, 85% and 80%, and were applied unchanged to the validation cohorts.

### 2.7. Clinical Utility and Model Comparison

Clinical utility was evaluated using decision curve analysis [26], across threshold probabilities of 0.01—0.60 for Model 1 and 0.20—0.95 for Model 2, reflecting the different outcome prevalence and clinically relevant decision ranges in the two target populations.

Each model was compared with the principal biochemical measurement available at its intended decision point: Model 1 with total calcium alone and Model 2 with PTH alone. Because serum calcium and PTH also contribute to the reference standard, these comparisons were interpreted as incremental discrimination relative to the corresponding biochemical measurement rather than as diagnostic accuracy against an independent reference standard. Model 2 was additionally compared with two previously published biochemical indices: the calcium-to-phosphate ratio [13] and the parathyroid function index (calcium × PTH/phosphate, with PTH expressed in pmol/L) [14]. Both indices were calculated as originally described and applied without refitting or optimisation in the present cohort. Paired AUCs were compared using the DeLong test. Because Model 1 is intended to support the same testing decision as an elevated calcium result, it was also compared with a total-calcium threshold at matched referral rates. At each Model 1 operating point, the calcium threshold was selected to refer the same proportion of participants. The proportions of PHPT cases identified by the two strategies were compared using McNemar’s test for paired, discordant outcomes.

### 2.8. Sensitivity Analyses

Three sensitivity analyses were conducted: (S1) excluding participants with total calcium above 3.5 or below 1.8 mmol/L; (S2) restricting the cohort to participants with eGFR ≥ 60 mL/min/1.73 m^2^; and (S3) restricting the cohort to participants with 25-hydroxyvitamin D ≥ 20 ng/mL.

Four additional exploratory analyses were conducted: S4 evaluated Model 2 among participants with PTH above 65 pg/mL and total calcium within the reference range, for whom serum calcium did not contribute to outcome classification; S5 compared participants with positive parathyroid histopathology with participants whose PTH normalised after vitamin D repletion and whose calcium remained within the reference range; S6 excluded hypercalcaemic participants classified as PHPT in whom familial hypocalciuric hypercalcaemia could not be excluded using the available clinical data, defined as PTH no greater than twice the upper reference limit, phosphate within the reference range and no history of nephrolithiasis. This definition was used only to identify a conservative exploratory phenotype and was not considered diagnostic of familial hypocalciuric hypercalcaemia; S7 evaluated Model 2 using alternative PTH thresholds of 65, 100 and 150 pg/mL to define subgroup eligibility.

All sensitivity analyses were conducted, where applicable, using the fixed model coefficients reported in Table 1 and without refitting.

### 2.9. Statistical Software and Reporting

Analyses were performed using Python (version 3.12.7) with pandas (version 2.2.2) for data management, NumPy (version 1.26.4) for numerical computation, scikit-learn (version 1.5.1) for multiple imputation, statsmodels (version 0.14.2) for model fitting and inference, SciPy (version 1.13.1) for statistical testing, and Matplotlib (version 3.9.2) for visualisation Continuous variables are summarised as mean ± standard deviation or median (interquartile range), as appropriate, and categorical variables as *n* (%). Between-group comparisons were performed using the Mann–Whitney U test for continuous variables and the χ^2^ test for categorical variables. All tests were two-sided, with two-sided *p* < 0.05 considered statistically significant. This study is reported in accordance with the TRIPOD+AI statement [18], and the completed checklist is provided in the Supplementary Materials.

## 3. Results

### 3.1. Cohort Characteristics

Of 5769 adults screened, 2788 met the eligibility criteria: 1843 at the Main campus (development cohort), 445 at East and 500 at Caotang (validation cohorts) (Figure 1). PHPT was adjudicated in 504 participants (18.1%), with prevalence differing across campuses (18.1%, 22.0% and 14.6%; *p* = 0.013) (Table 2). Participants at Caotang were older and had a higher prevalence of hypertension than those at the other campuses, indicating relevant case-mix heterogeneity.

Compared with participants without PHPT, those with PHPT were younger and had higher total calcium, PTH and alkaline phosphatase; lower phosphate and 25-hydroxyvitamin D; and more frequent nephrolithiasis (Table 3; Supplementary Tables S1 and S2).

Of the 2788 participants, 292 underwent parathyroid localisation imaging, of whom 252 had positive and 40 had negative findings. Five participants with negative imaging subsequently underwent ^18F-fluorocholine PET/CT, which was also negative. Eighty-eight participants underwent parathyroidectomy, and all had positive histopathology and were classified as PHPT. Localisation imaging was discordant with the final classification in 48 of 292 participants (16.4%): a total of 43 of 252 with positive imaging were classified as non-PHPT, whereas five of 40 with negative imaging were classified as PHPT on the basis of positive histopathology. Histopathology was fully concordant with the final classification.

### 3.2. Model 1—Before PTH Is Measured

**Figure 2:**
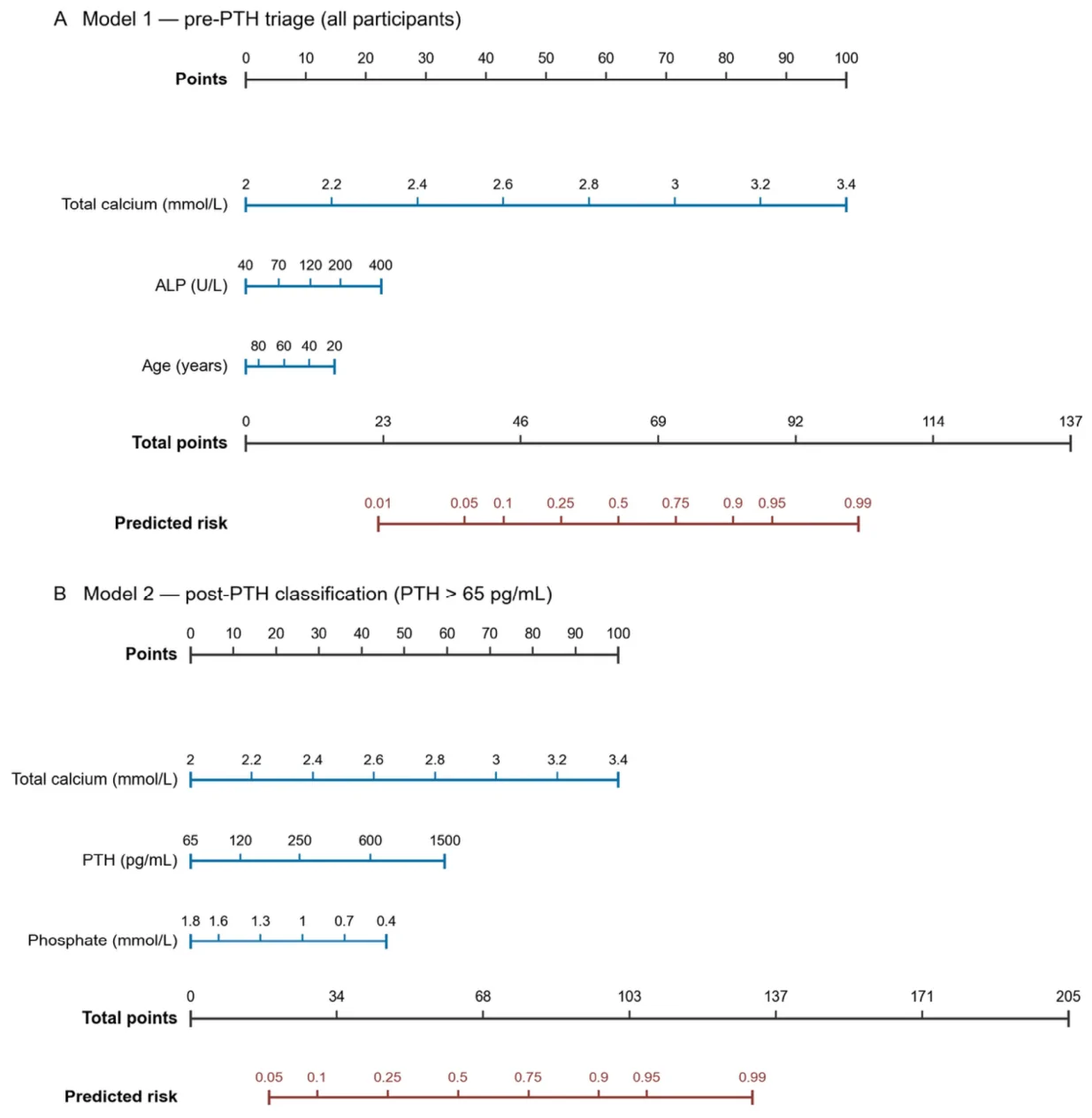
Nomograms for Model 1 and Model 2. Nomograms for (**A**) Model 1, based on total calcium, natural-log-transformed alkaline phosphatase and age, for use before PTH measurement; and (**B**) Model 2, based on natural-log-transformed PTH, total calcium and serum phosphate, for use in participants with PTH above the local upper reference limit of 65 pg/mL. For each predictor, the corresponding points are identified and summed; the total score is then mapped to the predicted probability of PHPT. ALP, alkaline phosphatase; PHPT, primary hyperparathyroidism; PTH, parathyroid hormone.

Model 1 comprised total calcium (odds ratio per standard deviation 9.78, 95% CI 7.55–12.68), ln(alkaline phosphatase) (1.62, 1.36–1.93) and age (0.71, 0.59–0.84); all *p* < 0.001 (Table 4, Figure 2A). The inverse association with age is consistent with the younger age distribution of participants with APH.

**Figure 3:**
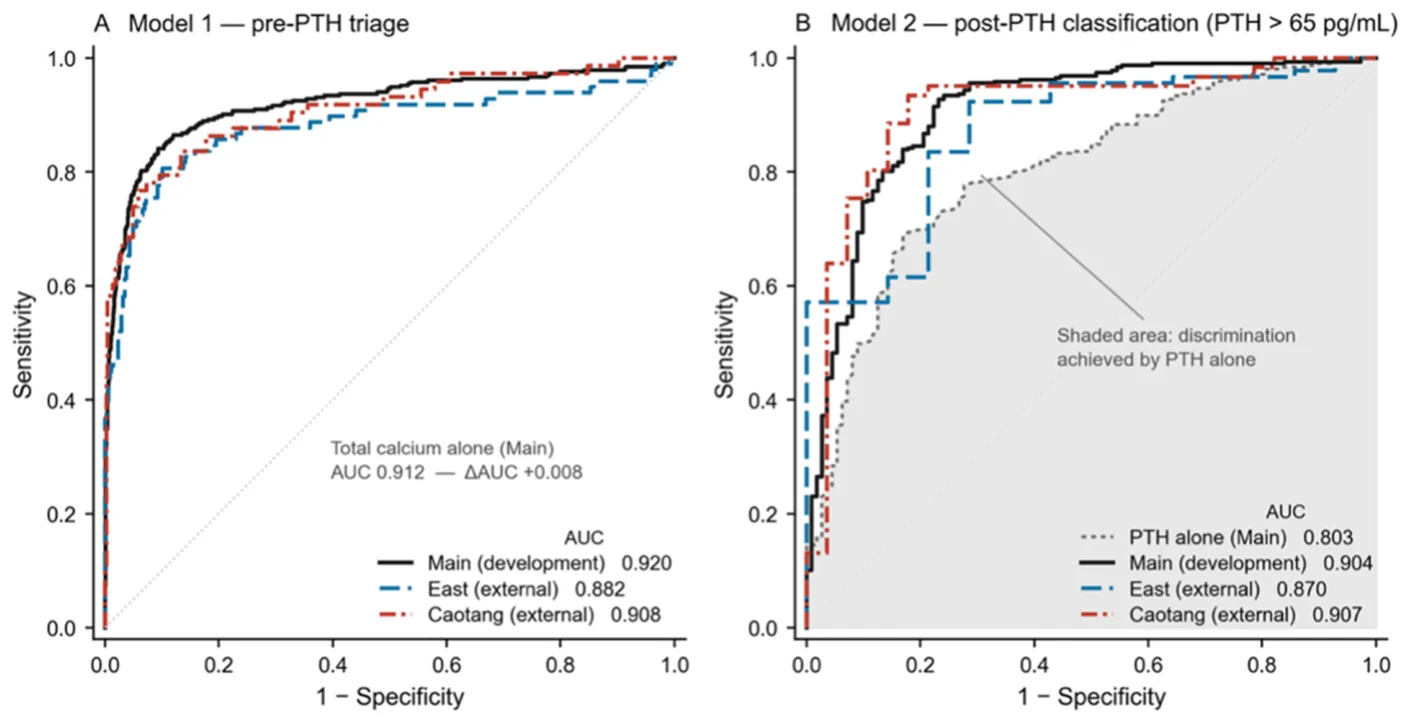
Discrimination of Model 1 and Model 2 relative to the single biochemical variable each is intended to supplement. Receiver operating characteristic curves for the development and validation cohorts, distinguished by colour and line style. (**A**) Model 1 in all participants, with total calcium alone shown for comparison in the development cohort. (**B**) Model 2 in participants with PTH above the local upper reference limit of 65 pg/mL, with PTH alone shown as a dashed line and the area under its curve shaded in the development cohort. Both models were fixed in the development cohort and applied to the validation cohorts without refitting. The diagonal line denotes chance performance. AUC, area under the receiver operating characteristic curve; PTH, parathyroid hormone.

**Figure 4:**
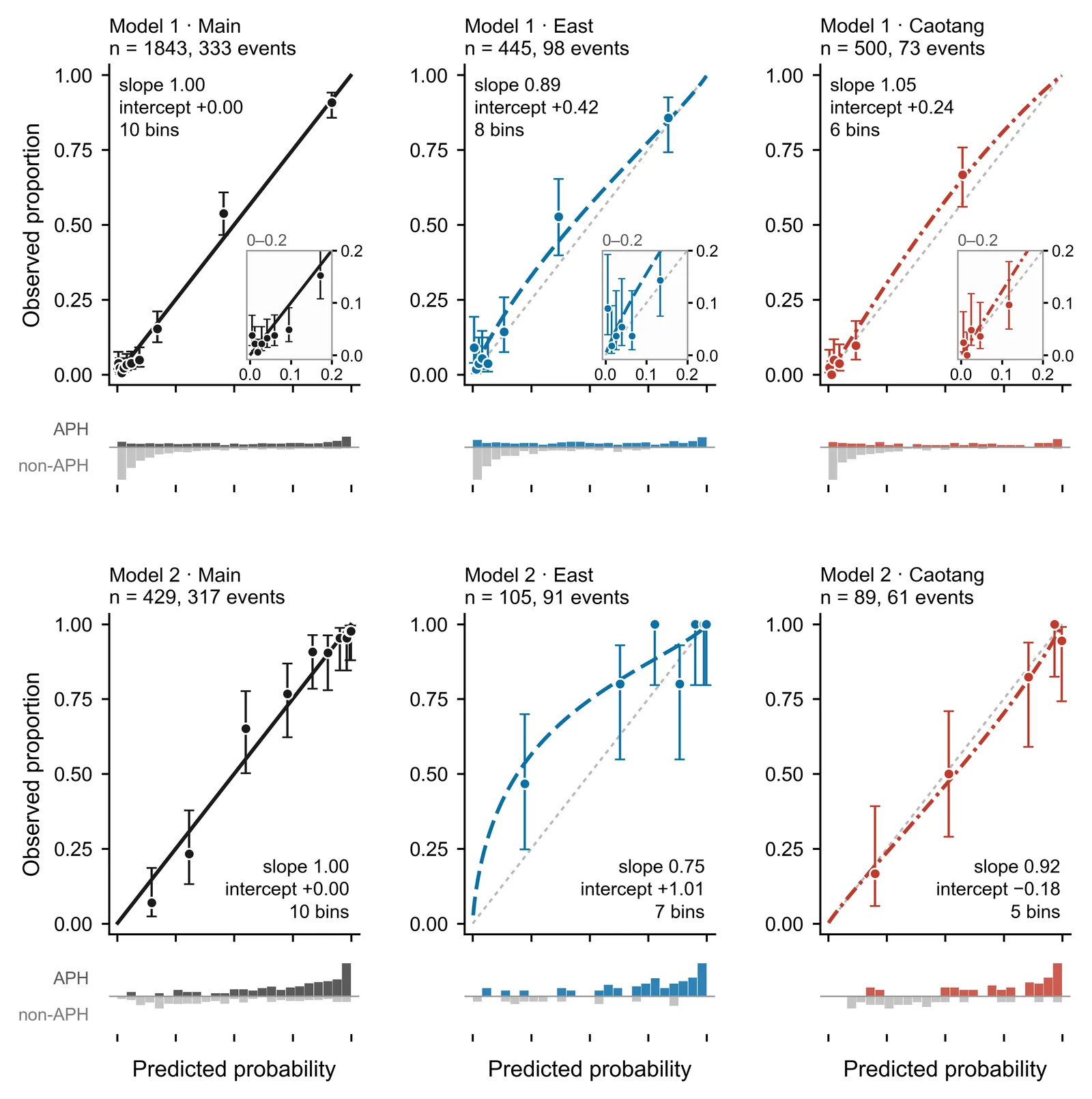
Calibration plots for Model 1 (**upper row**) and Model 2 (**lower row**) in the Main, East and Caotang cohorts. Points represent the observed PHPT proportion and mean predicted probability within quantile-based risk groups; error bars indicate 95% confidence intervals. The solid line represents the fitted calibration curve, and the dashed diagonal denotes perfect calibration. Insets magnify the predicted-probability range of 0–0.2 for Model 1. Mirrored histograms show the distribution of predicted probabilities on a square-root scale for participants with PHPT (above the axis) and without PHPT (below the axis). PHPT, primary hyperparathyroidism.

Because serum calcium contributed to the outcome definition, Model 1 performance reflects agreement with that definition rather than diagnostic accuracy against an independent reference standard. The AUC was 0.920 (95% CI 0.899–0.941) in the development cohort, with an optimism-corrected estimate of 0.920, and 0.882 (0.833–0.932) at East and 0.908 (0.863–0.953) at Caotang. Calibration varied across campuses, with systematic underprediction at East (calibration-in-the-large 0.42); complete performance statistics are reported in Table 5, Figure 3 and Figure 4.

**Figure 5:**
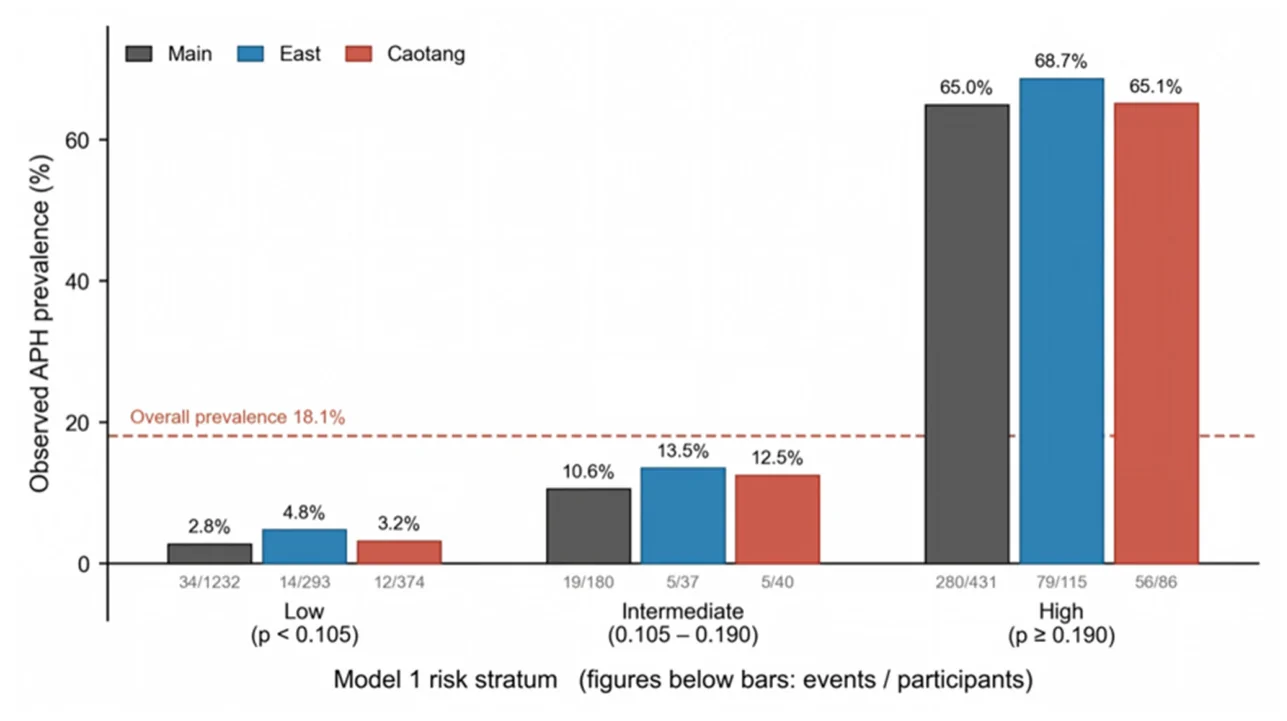
Risk stratification by Model 1. Observed PHPT prevalence across the low-, intermediate- and high-risk strata defined by Model 1 in each cohort. Predicted-probability thresholds of 0.105 and 0.190, corresponding to 90% sensitivity and 90% specificity in the Main campus development cohort, were applied unchanged to the East and Caotang validation cohorts. Bar heights represent observed PHPT prevalence, and labels beneath each bar show the number of PHPT cases and total participants within each stratum. The dashed horizontal line denotes the overall cohort prevalence of 18.1%. PHPT, primary hyperparathyroidism.

Compared with total calcium alone, Model 1 provided only modest and inconsistent improvements in discrimination (ΔAUC +0.002 to +0.008) (Table 6). At matched referral rates, total calcium identified 72.6–79.3% of PHPT cases, and Model 1 identified 75.5–80.2%. Using the development-cohort thresholds of 0.105 and 0.190, observed PHPT prevalence increased stepwise across the low-, intermediate- and high-risk groups, reaching 65.0–68.7% in the high-risk group; a total of 66–75% of each cohort was classified as low risk (Figure 5).

### 3.3. Model 2—After PTH Is Known to Be Elevated

Among 623 participants with PTH above 65 pg/mL, 469 (75.3%) were adjudicated as PHPT. PTH alone achieved AUCs of 0.803, 0.811 and 0.793 at Main, East and Caotang, respectively.

Model 2 comprised total calcium (odds ratio per standard deviation 6.21, 95% CI 3.80–10.13; *p* < 0.001), ln(PTH) (2.64, 1.52–4.57; *p* < 0.001) and phosphate (0.61, 0.43–0.87; *p* = 0.006) (Table 4, Figure 2B). The AUC was 0.904 (0.869–0.940) in the development cohort, with an optimism-corrected estimate of 0.900. In the pooled validation cohort (194 participants, 152 events and 42 non-events), Model 2 achieved an AUC of 0.892 (0.829–0.945) compared with 0.791 for PTH alone (ΔAUC +0.101, *p* = 0.002), with a calibration slope of 0.88. The East cohort contained only 14 participants without PHPT; its campus-specific incremental and calibration estimates are therefore reported in the Supplementary Materials.

Model 2 showed higher discrimination than the calcium-to-phosphate ratio and parathyroid function index, although the differences were modest and not consistently statistically significant across campuses (Table 6). Complete campus-specific performance estimates are provided in Table 6 and Table 7.

### 3.4. Model 2 in Participants with Elevated PTH and Normal Calcium

Model 2 was additionally evaluated in 214 participants with PTH above 65 pg/mL and total calcium within the reference range, including 83 PHPT cases. In this subgroup, serum calcium did not contribute to outcome classification. Using the fixed model coefficients without refitting, Model 2 achieved an AUC of 0.790 overall compared with 0.699 for PTH alone. Campus-specific AUCs were 0.817 at Main, 0.704 at East and 0.745 at Caotang. Discrimination was lower than in the full PTH-elevated population, and no incremental value over PTH alone was observed at East.

### 3.5. Sequential Use of the Two Models

At the Model 1 operating point corresponding to 85% sensitivity in the development cohort (predicted probability ≥ 0.181), 18.0–26.1% of participants across campuses would proceed to PTH and phosphate measurement, identifying 78.1–85.0% of PHPT cases. Among those referred, 299 participants at Main, 82 at East and 58 at Caotang had PTH above 65 pg/mL and would proceed to Model 2. These second-stage subgroups had an observed PHPT prevalence of 93.1–95.1%, compared with 18.1% in the full cohort (Table 8). Results for the 90%- and 80%-sensitivity operating points are also provided in Table 8.

**Table 8:** Performance of the two-stage cascade.

Operating Point	Cohort	Referred (%)	PHPT Captured (%)	Entering Model 2 (*n*)	PHPT Prevalence in Model 2 Population (%)
Model 1 sensitivity 90% (*p* ≥ 0.105)	Main	33.2	89.8	326	89.9
Model 1 sensitivity 90% (*p* ≥ 0.105)	East	34.2	85.7	89	93.3
Model 1 sensitivity 90% (*p* ≥ 0.105)	Caotang	25.2	83.6	60	93.3
Model 1 sensitivity 85% (*p* ≥ 0.181)	Main	24.0	85.0	299	93.3
Model 1 sensitivity 85% (*p* ≥ 0.181)	East	26.1	80.6	82	95.1
Model 1 sensitivity 85% (*p* ≥ 0.181)	Caotang	18.0	78.1	58	93.1
Model 1 sensitivity 80% (*p* ≥ 0.269)	Main	19.6	79.9	279	94.3
Model 1 sensitivity 80% (*p* ≥ 0.269)	East	21.8	74.5	74	97.3
Model 1 sensitivity 80% (*p* ≥ 0.269)	Caotang	12.6	67.1	51	92.2

Three Model 1 operating points were derived in the development cohort to correspond to sensitivities of 90%, 85% and 80% and were applied unchanged to the East and Caotang validation cohorts. “Referred” denotes participants for whom Model 1 would recommend PTH and phosphate measurement; “entering Model 2” denotes referred participants with PTH above the local upper reference limit of 65 pg/mL. PHPT prevalence in the full analysis cohort was 18.1%. These development-cohort thresholds require prospective validation and, where necessary, local adaptation before clinical implementation. Abbreviations are defined in Table 2.

### 3.6. Clinical Utility

**Figure 6:**
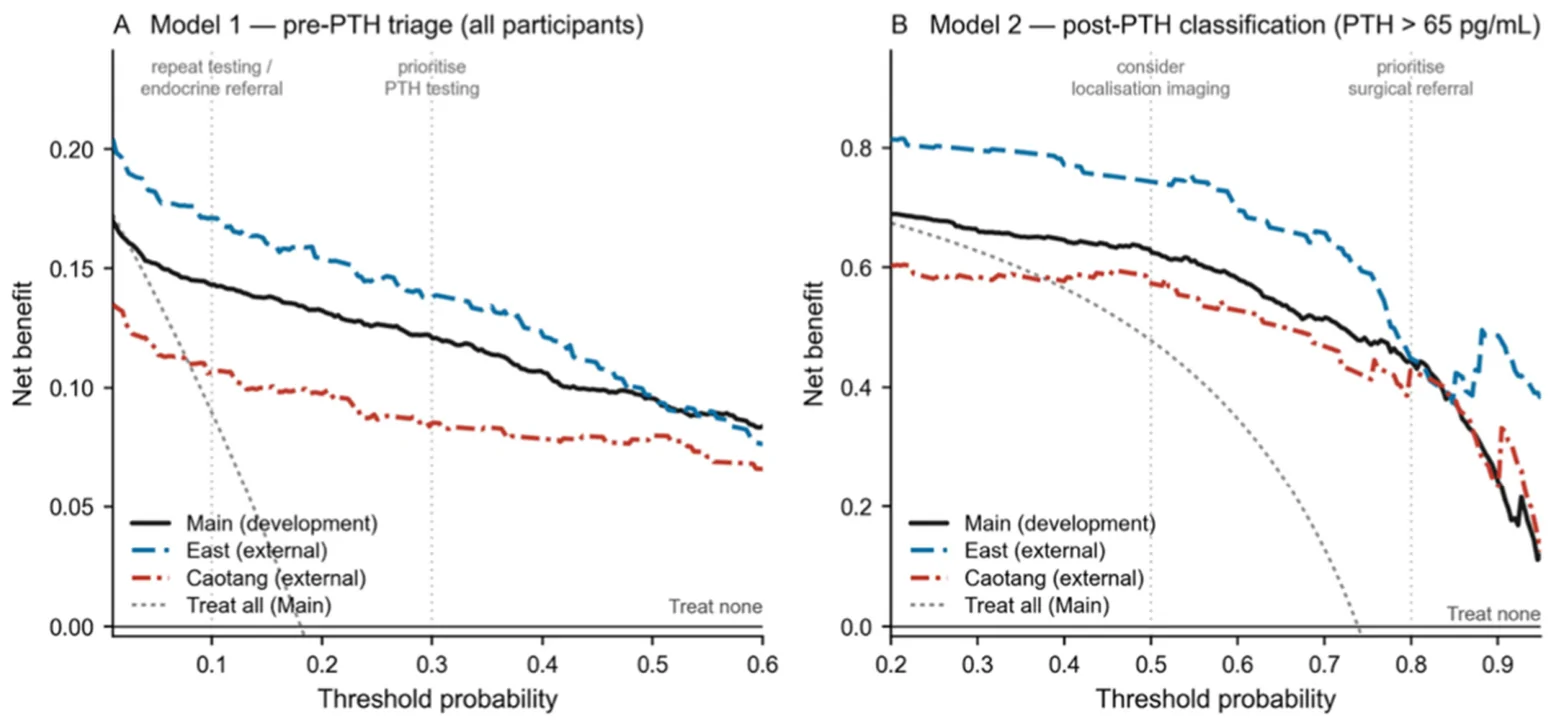
Decision curve analysis. Decision curve analysis for (**A**) Model 1 in all participants and (**B**) Model 2 in participants with PTH above the local upper reference limit of 65 pg/mL. Each model was compared with evaluate-all and evaluate-none strategies across threshold probabilities of 0.01–0.60 for Model 1 and 0.20–0.95 for Model 2. The vertical-axis scales differ between panels. The horizontal line at zero denotes the evaluate-none strategy, and the dashed line denotes the evaluate-all strategy in the development cohort. Dotted vertical lines indicate the evaluated clinical operating thresholds. PTH, parathyroid hormone.

Decision curve analysis showed a positive net benefit for Model 1 relative to evaluate-all and evaluate-none strategies across threshold probabilities of 0.01–0.60 in all three cohorts and for Model 2 across threshold probabilities of 0.20–0.95 (Figure 6).

### 3.7. Sensitivity Analyses

Sensitivity analyses were broadly consistent with the primary findings (Table 9). Excluding extreme calcium values or restricting the analysis to participants with eGFR ≥ 60 mL/min/1.73 m^2^ changed AUCs by no more than 0.030. Analyses restricted to participants with 25-hydroxyvitamin D ≥ 20 ng/mL were based on substantially smaller samples and yielded imprecise estimates.

Excluding 74 participants in whom familial hypocalciuric hypercalcaemia could not be excluded changed AUCs by no more than 0.023. Increasing the PTH threshold for Model 2 eligibility from 65 to 150 pg/mL reduced the AUC from 0.900 to 0.771. In the focused comparison of 88 histopathology-positive cases and 131 participants whose PTH normalised after vitamin D repletion and whose calcium remained within the reference range, Model 1 achieved an AUC of 0.907 compared with 0.902 for total calcium alone, and Model 2 achieved an AUC of 0.922 compared with 0.863 for PTH alone (Supplementary Table S6). Because both groups were highly selected, these estimates should not be interpreted as performance in a routine clinical population. Detailed sensitivity-analysis results are provided in Table 9 and the Supplementary Materials.

## 4. Discussion

In this multicentre retrospective study, we developed two interpretable models corresponding to distinct decision points in the evaluation of suspected PHPT and assessed them using an internal–external validation design. The models provided different information at the two decision points. Model 1 largely formalised an existing calcium-based testing rule, whereas Model 2 provided additional discrimination after PTH elevation had been identified.

Among participants with elevated PTH, the calcium–phosphate phenotype appeared to provide information beyond the PTH concentration alone. Model 2 showed higher discrimination than PTH alone in the development and pooled validation cohorts. However, because serum calcium contributed to outcome classification in hypercalcaemic participants, performance in the full PTH-elevated population may have been affected by incorporation bias. In the normocalcaemic subgroup, in which serum calcium did not contribute to outcome classification, Model 2 achieved an AUC of 0.790 compared with 0.699 for PTH alone. Performance varied across campuses, and no incremental value was observed at East, so this result should be regarded as supportive rather than definitive.

The contribution of calcium and phosphate is biologically plausible. Once a population has been selected on the basis of elevated PTH, further variation in PTH concentration may provide limited additional information. Calcium and phosphate reflect the downstream renal and skeletal effects of PTH, and hypophosphataemia in the presence of relatively high calcium is consistent with autonomous PTH secretion. These findings support the proposed pathophysiological rationale but do not establish the underlying mechanism.

Several published biochemical indices encode a similar calcium–phosphate phenotype, including the calcium-to-phosphate ratio [13], the parathyroid function index [14] and the calcium × chloride/phosphate ratio [27]. The two indices evaluated in the present study performed reasonably well, consistent with previous reports [28,29]. Model 2 showed modestly higher discrimination, but the differences were not consistently statistically significant across campuses. Its potential practical advantage is that it provides an individual predicted probability that can be evaluated and, where necessary, updated locally. Nevertheless, the comparison group in the present study was also largely vitamin D deficient. We therefore cannot conclude that Model 2 overcomes the limitations previously identified for these biochemical indices.

Before PTH measurement, Model 1 provided little incremental discrimination over total calcium alone and should not be regarded as a superior diagnostic model. Its potential value lies in providing an explicit, reproducible and auditable representation of a calcium-based testing rule. At matched referral rates, Model 1 and a total-calcium threshold identified similar proportions of PHPT cases. When the two models were applied sequentially, Model 1 selected 18–26% of participants for further testing while identifying 78–85% of PHPT cases. Model 2 was then applied to participants with elevated PTH, identifying second-stage subgroups with an observed PHPT prevalence of 93–95%. These results describe enrichment within a selected cohort rather than an increase in disease prevalence or evidence of clinical effectiveness.

Previous prediction studies in hyperparathyroidism have largely focused on surgical cohorts, diagnostic classification or preoperative localisation [30,31,32]. One single-centre study identified patients at risk of PHPT without using calcium or PTH but did not report external validation or calibration [33]. In contrast, the present models were designed for two earlier clinical decision points and use three routinely available variables each. Their simplicity and complete reporting may facilitate independent evaluation and potential implementation in laboratory information systems, although clinical use would require further validation.

Several potential sources of outcome misclassification remain. Hypercalciuria-related secondary hyperparathyroidism and disorders of vitamin D metabolism could have been misclassified as PHPT, primarily among the 83 participants with normal calcium and persistently elevated PTH who were classified as cases. These participants represented 16.5% of PHPT cases and 3.0% of the analysis cohort. Positive localisation imaging or histopathology in 41 of these participants supported the diagnosis but did not exclude secondary causes. Although dynamic diagnostic protocols—such as the hydrochlorothiazide challenge test for idiopathic hypercalciuria [34,35] and short suppression tests with active vitamin D metabolites [36]—have been proposed to assist in differentiating normocalcaemic PHPT from secondary hyperparathyroidism, these procedures are time-consuming or inpatient-oriented and were not routinely feasible in our retrospective outpatient setting. Familial hypocalciuric hypercalcaemia could not be systematically excluded because urinary calcium, the calcium-to-creatinine clearance ratio, family history and CASR genotyping were unavailable. An inappropriately normal PTH concentration in the presence of hypercalcaemia is a recognised biochemical presentation of PHPT; the principal concern in these participants is that calcium contributed to both outcome classification and Model 1. Finally, because participants with malignancy were excluded, neither model was evaluated for differentiating PHPT from hypercalcaemia of malignancy. The models are therefore intended for outpatient assessment and not for determining the cause of hypercalcaemia in hospitalised patients.

## 5. Limitations

Several limitations should be considered. First, all participants had already undergone PTH testing. Model 1 is framed as informing whether PTH should be measured, but a cohort containing no untested patients cannot establish how such a rule would perform at that decision point; it can show only how the model behaves among patients a clinician had already elected to test. Eligibility also required more than 80% completeness across the collected clinical variables, and completeness is itself related to the degree of clinical suspicion. The cohort is therefore enriched for suspected disease, and both discrimination and positive predictive value would be expected to be lower in an unselected outpatient population. For the same reason, this cohort cannot quantify how often an elevated calcium goes without PTH measurement in routine care—the gap the pre-PTH step is intended to address. Second, although the three campuses are geographically distinct and served as separate development and validation cohorts, they share an electronic record system and laboratory service; calcium and PTH assays were not formally harmonised across sites. The analysis is therefore best described as internal–external rather than fully independent external validation [24], and independent replication in an unrelated healthcare system remains necessary. Third, within the PTH-elevated subgroup, the East cohort contained only 14 participants without PHPT. All Model 2 estimates from that cohort—discrimination, calibration and the comparisons against published indices—are correspondingly imprecise and should be interpreted with caution; the pooled external cohort contains 42 non-events and is also insufficient, so Model 2 requires validation in a population with adequate numbers of both events and non-events. Finally, the risk thresholds were derived in the development cohort. Although they transferred without modification to both validation cohorts, the threshold of 65 pg/mL that defines eligibility for Model 2 is the local reference upper limit of a second-generation assay and is not a general threshold. Before Model 2 is applied on a different platform or assay generation, the target population must be redefined from the local reference interval and the model independently validated, with model updating where required, rather than recalibrated alone. The exclusion of the 875 participants with more than 20% missing data may itself have introduced selection bias, since the extent of missingness is related to the degree of clinical suspicion, and it further enriches the cohort relative to routine practice. Urinary calcium, the calcium-to-creatinine clearance ratio, family history and CASR genotyping were not available, so familial hypocalciuric hypercalcaemia could not be systematically excluded, and some participants classified as cases could have that condition. Histopathological confirmation and post-operative biochemical remission were not available for all participants, which is a further limitation of the reference standard. The age coefficient in Model 1 may reflect local testing and referral practice, and the model cannot be assumed to transport to settings in which the relationship between age, calcium testing and referral differs. Because malignancy and treatments affecting calcium or bone metabolism were excluded at enrolment, the cohort is highly selected, and both models are intended for outpatient evaluation rather than for the investigation of hypercalcaemia in hospitalised patients.

## 6. Conclusions

This study developed and evaluated a two-stage strategy for supporting PHPT testing and referral using an internal–external validation design. The pre-PTH model formalised an existing calcium-based testing rule, whereas the post-PTH model showed additional discrimination beyond PTH alone, including in the pooled normocalcaemic subgroup. Both models use routinely available measurements but require prospective validation in broader, less selected populations before clinical implementation.

## Figures and Tables

**Table 1:** Model equations.

Model	Equation
Model 1—pre-PTH triage (development cohort *n* = 1843)	logit(*p*) = −25.5181 + 8.2136 × total calcium (mmol/L) + 1.1250 × ln[ALP (U/L)] − 0.0243 × age (years)
Model 2—post-PTH classification (development cohort *n* = 429; PTH > 65 pg/mL)	logit(*p*) = −15.0533 + 1.2642 × ln[PTH (pg/mL)] + 4.7667 × total calcium (mmol/L) − 2.1827 × phosphate (mmol/L)

Coefficients are on the original measurement scale and may be applied directly. Predicted probability is obtained as *p* = 1/(1 + e^(−logit)^).

**Table 2:** Baseline characteristics of the analysis cohort by campus.

Variable	Overall (*n* = 2788)	Main (*n* = 1843)	East (*n* = 445)	Caotang (*n* = 500)	*p* Value
**Outcome**					
PHPT, *n* (%)	504 (18.1)	333 (18.1)	98 (22.0)	73 (14.6)	0.013
**Demographic characteristics**					
Age (years)	59.2 ± 14.3	57.4 ± 13.8	57.5 ± 14.1	67.6 ± 13.2	<0.001
Male, *n* (%)	727 (26.1)	482 (26.2)	109 (24.5)	136 (27.2)	0.634
BMI (kg/m^2^)	23.5 ± 3.5	23.5 ± 3.6	23.5 ± 3.8	23.6 ± 3.3	0.464
**Comorbidities**					
Hypertension, *n* (%)	991 (35.5)	607 (32.9)	155 (34.8)	229 (45.8)	<0.001
Osteoporosis, *n* (%)	1171 (42.0)	718 (39.0)	179 (40.2)	274 (54.8)	<0.001
Nephrolithiasis, *n* (%)	323 (11.6)	232 (12.6)	42 (9.4)	49 (9.8)	0.068
**Calcium–phosphate metabolism**					
PTH (pg/mL)	43.8 (31.5–61.3)	43.8 (31.3–62.3)	45.2 (32.3–62.8)	43.2 (31.2–57.9)	0.231
Total calcium (mmol/L)	2.37 ± 0.28	2.38 ± 0.28	2.38 ± 0.28	2.35 ± 0.26	0.058
Albumin-corrected calcium (mmol/L)	2.36 ± 0.26	2.36 ± 0.26	2.37 ± 0.25	2.34 ± 0.24	0.105
Phosphate (mmol/L)	1.10 ± 0.25	1.10 ± 0.25	1.09 ± 0.27	1.10 ± 0.23	0.869
Magnesium (mmol/L)	0.88 ± 0.10	0.88 ± 0.10	0.87 ± 0.10	0.89 ± 0.10	0.064
ALP (U/L)	83.0 (66.0–106.2)	83.0 (67.0–108.0)	81.3 (64.0–106.2)	82.0 (66.0–103.2)	0.252
Albumin (g/L)	40.9 ± 4.4	41.0 ± 4.4	40.8 ± 4.5	40.6 ± 4.2	0.019
**Bone turnover and vitamin D**					
25(OH)D (ng/mL)	16.9 (12.4–22.8)	17.0 (12.4–23.0)	16.3 (12.0–21.4)	17.2 (13.0–23.1)	0.100
P1NP (ng/mL)	53.1 (35.4–78.6)	54.3 (35.6–81.5)	52.7 (36.5–77.3)	49.3 (33.1–72.3)	0.006
β-CTX (pg/mL)	487.9 (284.0–761.0)	498.5 (297.8–779.5)	498.4 (268.5–782.0)	413.8 (260.9–666.6)	<0.001
**Renal function and other indices**					
eGFR (mL/min/1.73 m^2^)	90.9 ± 22.5	93.4 ± 21.0	93.5 ± 22.0	79.4 ± 24.9	<0.001
Creatinine (μmol/L)	60.7 (51.5–76.4)	59.9 (51.2–73.7)	59.2 (50.6–74.2)	68.1 (53.6–91.8)	<0.001
Urea (mmol/L)	5.6 (4.5–7.0)	5.5 (4.4–6.8)	5.4 (4.4–6.8)	6.2 (4.9–8.1)	<0.001
Uric acid (μmol/L)	310.0 (255.0–373.0)	303.0 (251.0–368.0)	312.5 (255.0–384.0)	323.0 (267.8–385.2)	<0.001
Haemoglobin (g/L)	128.4 ± 17.7	129.1 ± 17.4	128.1 ± 17.2	126.4 ± 18.9	0.025
ALT (U/L)	19.0 (13.0–28.0)	19.0 (13.0–29.0)	19.0 (13.0–28.0)	17.0 (13.0–26.0)	0.003
AST (U/L)	23.0 (21.0–27.0)	23.0 (21.0–27.0)	23.0 (21.0–27.0)	23.0 (21.0–26.2)	0.080

Values are mean ± standard deviation for continuous variables and *n* (%) for categorical variables, except for skewed variables (PTH, ALP, creatinine, urea, uric acid, 25(OH)D, P1NP, β-CTX, ALT, AST), which are given as median (interquartile range). *p* values are from one-way analysis of variance or the Kruskal–Wallis test for continuous variables and the χ^2^ test for categorical variables. The Main campus served as the development cohort, and the East and Caotang campuses served as external validation cohorts. Participants at the Caotang campus were approximately ten years older than those at the other two campuses (67.6 versus 57.4 and 57.5 years), and observed APH prevalence differed across campuses (18.1%, 22.0% and 14.6%); both differences are relevant to the interpretation of between-cohort differences in model performance. Missing data ranged from 0% to 6.5% and are reported by variable and outcome status in Supplementary Table S3. ALP, alkaline phosphatase; ALT, alanine aminotransferase; APH, autonomous parathyroid hormone hypersecretion; AST, aspartate aminotransferase; BMI, body mass index; eGFR, estimated glomerular filtration rate; PTH, parathyroid hormone; 25(OH)D, 25-hydroxyvitamin D; P1NP, procollagen type I *n*-terminal propeptide; β-CTX, β-C-terminal telopeptide of type I collagen.

**Table 3:** Baseline characteristics of the analysis cohort by PHPT status.

Variable	Overall (*n* = 2788)	Non-APH (*n* = 2284)	APH (*n* = 504)	*p* Value
**Demographic characteristics**				
Age (years)	59.2 ± 14.3	60.5 ± 14.1	53.4 ± 13.8	<0.001
Male, *n* (%)	727 (26.1)	582 (25.5)	145 (28.8)	0.143
BMI (kg/m^2^)	23.5 ± 3.5	23.6 ± 3.6	23.1 ± 3.2	0.033
**Comorbidities**				
Hypertension, *n* (%)	991 (35.5)	851 (37.3)	140 (27.8)	<0.001
Osteoporosis, *n* (%)	1171 (42.0)	1027 (45.0)	144 (28.6)	<0.001
Nephrolithiasis, *n* (%)	323 (11.6)	170 (7.4)	153 (30.4)	<0.001
**Calcium–phosphate metabolism and vitamin D**				
PTH (pg/mL)	43.8 (31.5–61.3)	39.7 (29.4–51.2)	162.0 (99.5–290.2)	<0.001
Total calcium (mmol/L)	2.37 ± 0.28	2.29 ± 0.16	2.76 ± 0.35	<0.001
Albumin-corrected calcium (mmol/L)	2.36 ± 0.26	2.28 ± 0.14	2.71 ± 0.35	<0.001
Phosphate (mmol/L)	1.10 ± 0.25	1.15 ± 0.22	0.84 ± 0.21	<0.001
ALP (U/L)	83.0 (66.0–106.2)	79.4 (65.0–99.0)	109.0 (79.0–152.0)	<0.001
Albumin (g/L)	40.9 ± 4.4	40.6 ± 4.4	42.2 ± 4.0	<0.001
25(OH)D (ng/mL)	16.9 (12.4–22.8)	17.7 (13.0–23.5)	13.8 (9.8–17.6)	<0.001
**Renal function**				
eGFR (mL/min/1.73 m^2^)	90.9 ± 22.5	90.4 ± 22.2	93.0 ± 24.1	0.027

Values are presented as mean ± standard deviation, median (interquartile range) for skewed variables (PTH, ALP and 25(OH)D), or *n* (%), as appropriate. *p* values were calculated using the independent-samples *t* test for normally distributed continuous variables, the Mann–Whitney U test for skewed continuous variables, and the χ^2^ test for categorical variables. Variables relevant to model development and interpretation are shown; the complete baseline characteristics are provided in Supplementary Table S1. Abbreviations are defined in Table 2.

**Table 4:** Multivariable logistic regression models for PHPT.

Model	Variable	β Coefficient	SD of Predictor	OR per 1 SD	95% CI	*p* Value
Model 1	Intercept	−25.5181				
Model 1	Total calcium (mmol/L)	8.2136	0.278	9.78	7.55–12.68	<0.001
Model 1	ln[ALP (U/L)]	1.125	0.429	1.62	1.36–1.93	<0.001
Model 1	Age (years)	−0.0243	14.287	0.71	0.59–0.84	<0.001
Model 2	Intercept	−15.0533				
Model 2	ln[PTH (pg/mL)]	1.2642	0.768	2.64	1.52–4.57	<0.001
Model 2	Total calcium (mmol/L)	4.7667	0.383	6.21	3.80–10.13	<0.001
Model 2	Phosphate (mmol/L)	−2.1827	0.226	0.61	0.43–0.87	0.006

Model coefficients were estimated in the Main campus development cohort and pooled across 10 multiply imputed datasets using Rubin’s rules. Odds ratios are reported per one-standard-deviation increase in each predictor; the corresponding standard deviations are provided in the table. Model 1 was developed in all 1843 participants for use before PTH measurement. Model 2 was developed in the 429 participants with PTH above the local upper reference limit of 65 pg/mL for use after PTH elevation had been identified. ALP and PTH were natural-log transformed. Abbreviations are defined in Table 2; CI, confidence interval; OR, odds ratio; SD, standard deviation.

**Table 5:** Discrimination and calibration of Model 1 and Model 2.

Model	Cohort	*n*	Events	AUC	95% CI	Optimism-Corrected AUC	Brier Score	Calibration Slope	Calibration Intercept
Model 1	Main (development)	1843	333	0.920	0.899–0.941	0.920	0.0645	1.00	0.00
Model 1	East (external)	445	98	0.882	0.833–0.932		0.0862	0.89	0.42
Model 1	Caotang (external)	500	73	0.908	0.863–0.953		0.0592	1.05	0.24
Model 2	Main (development)	429	317	0.904	0.869–0.940	0.900	0.0971	1.00	0.00
Model 2	East (external)	105	91	0.870	0.778–0.963		0.0998	0.75	1.01
Model 2	Caotang (external)	89	61	0.907	0.831–0.982		0.1025	0.92	−0.18
Model 2	East + Caotang (pooled external)	194	152	0.892	0.892–0.945		0.1010	0.88	0.38
Reference: PTH alone	Main (development)	429	317	0.803	0.757–0.849				
Reference: PTH alone	East (external)	105	91	0.811	0.696–0.926				
Reference: PTH alone	Caotang (external)	89	61	0.793	0.701–0.885				

Both models were fixed in the Main campus development cohort and applied to the East and Caotang validation cohorts without refitting. AUCs are reported with DeLong 95% confidence intervals. Calibration was assessed using the calibration slope and calibration-in-the-large, and overall performance was assessed using the Brier score. Optimism-corrected estimates for the development cohort were obtained using bootstrap resampling with 200 replications and differed from the apparent estimates by no more than 0.004, suggesting limited apparent optimism. Model 2 was evaluated only in participants with PTH above the local upper reference limit of 65 pg/mL, accounting for the smaller sample sizes. Because the East cohort contained only 14 participants without PHPT in this subgroup, its campus-specific estimates should be interpreted cautiously, and its calibration statistics are reported in Supplementary Table S5. The pooled East and Caotang estimate is therefore presented as the principal validation result for Model 2. AUC, area under the receiver operating characteristic curve; CI, confidence interval; PTH, parathyroid hormone.

**Table 6:** Incremental discriminative value of each model over the single biochemical variable it is intended to supplement.

Comparison	Cohort	Model AUC	Reference AUC	ΔAUC	*p* Value (DeLong)
Model 1 vs. total calcium alone	Main	0.92	0.912	+0.008	0.003
Model 1 vs. total calcium alone	East	0.882	0.88	+0.002	0.651
Model 1 vs. total calcium alone	Caotang	0.908	0.902	+0.006	0.307
Model 2 vs. PTH alone	Main	0.904	0.803	+0.101	<0.001
Model 2 vs. PTH alone	East	0.87	0.811	+0.060	0.231
Model 2 vs. PTH alone	Caotang	0.907	0.793	+0.114	0.003
Model 2 vs. PTH alone	East + Caotang (pooled)	0.892	0.791	+0.101	0.002

Each model was compared with the principal biochemical measurement available at its intended decision point: Model 1 with total calcium alone in all participants and Model 2 with PTH alone among participants with PTH above the local upper reference limit of 65 pg/mL. Because serum calcium and PTH also contributed to the reference standard, these comparisons represent incremental discrimination relative to the corresponding biochemical measurement rather than diagnostic accuracy against an independent reference standard. Paired AUCs were compared using the DeLong test. Comparisons between Model 2 and previously published biochemical indices are reported in Table 7. Because the East cohort included only 14 participants without PHPT in the Model 2 subgroup, its campus-specific comparison is reported in Supplementary Table S5, and the pooled East and Caotang comparison is presented as the principal validation result. AUC, area under the receiver operating characteristic curve; PTH, parathyroid hormone.

**Table 7:** Comparison of Model 2 with previously published biochemical indices.

Index or Model	Main (*n* = 429)	East (*n* = 105)	Caotang (*n* = 89)	ΔAUC vs. Model 2, Main (*p*)	ΔAUC vs. Model 2, East (*p*)	ΔAUC vs. Model 2, Caotang (*p*)
PTH alone	0.803 (0.757–0.849)	0.811 (0.696–0.926)	0.793 (0.701–0.885)	+0.101 (<0.001)	+0.060 (0.231)	+0.114 (0.003)
Calcium/phosphate ratio (Madeo et al.)	0.858 (0.815–0.902)	0.801 (0.688–0.915)	0.886 (0.807–0.965)	+0.046 (<0.001)	+0.069 (0.032)	+0.021 (0.238)
Parathyroid function index, Ca × PTH/P (Guo et al.)	0.866 (0.825–0.907)	0.828 (0.716–0.940)	0.858 (0.779–0.938)	+0.039 (0.004)	+0.042 (0.247)	+0.049 (0.041)
Model 2 (present study)	0.904 (0.869–0.940)	0.870 (0.778–0.963)	0.907 (0.831–0.982)	—	—	—

Model 2 was compared with previously published biochemical indices among participants with PTH above the local upper reference limit of 65 pg/mL. The calcium-to-phosphate ratio and parathyroid function index (calcium × PTH/phosphate, with PTH expressed in pmol/L) were calculated as originally described and applied without refitting or optimisation. AUCs are reported with DeLong 95% confidence intervals. ΔAUC represents the difference between Model 2 and each comparator; corresponding *p* values were calculated using the DeLong test. AUC, area under the receiver operating characteristic curve; PTH, parathyroid hormone.

**Table 9:** Sensitivity analyses.

Model	Analysis	*n*	Events	Main	East	Caotang
Model 1	Primary analysis	2788	504	0.920	0.882	0.908
Model 2	Primary analysis	623	469	0.904	0.870	0.907
Model 1	S1—excluding total calcium >3.5 or <1.8 mmol/L	2760	487	0.916	0.888	0.906
Model 2	S1—excluding total calcium >3.5 or <1.8 mmol/L	604	452	0.899	0.867	0.905
Model 1	S2—restricted to eGFR ≥ 60 mL/min/1.73 m^2^	2448	443	0.918	0.877	0.896
Model 2	S2 —restricted to eGFR ≥ 60 mL/min/1.73 m^2^	549	413	0.920	0.867	0.936
Model 1	S3—restricted to 25(OH)D ≥ 20 ng/mL	968	87	0.885	0.773	0.884
Model 2	S3—restricted to 25(OH)D ≥ 20 ng/mL	127	71	0.903	0.844	0.896
Model 2	S4—PTH > 65 pg/mL and calcium within reference range	214	83	0.817	0.704	0.745
Model 1	S6—excluding participants with FHH-compatible biochemistry	2714	430	0.911	0.859	0.902
Model 2	S6—excluding participants with FHH-compatible biochemistry	553	399	0.910	0.889	0.911

Values are AUCs averaged across 10 imputed datasets. S1 excluded participants with total calcium above 3.5 or below 1.8 mmol/L. S2 restricted the analysis to participants with eGFR ≥ 60 mL/min/1.73 m^2^. S3 restricted the analysis to participants with 25-hydroxyvitamin D ≥ 20 ng/mL, resulting in 968 participants (87 events) for Model 1 and 127 participants (71 events) for Model 2. S4 evaluated Model 2 among participants with PTH above the local upper reference limit of 65 pg/mL and total calcium within the reference range. S6 excluded participants in whom familial hypocalciuric hypercalcaemia could not be excluded using the available clinical and biochemical data. All analyses used the fixed model coefficients without refitting. Abbreviations are defined in Table 2.

## Data Availability

The data presented in this study are available on request from the corresponding author due to restrictions related to patient privacy and the terms of the Institutional Review Board approval, which do not permit public deposition of individual-level clinical data. The code used to generate all tables and figures is available from the corresponding authors upon request. The complete model coefficients and the unit conventions required to apply both models are provided in Table 1, allowing the models to be reproduced and applied without access to the underlying data.

## Supplementary Materials

The following supporting information can be downloaded at: https://www.mdpi.com/article/10.3390/diagnostics16193200/s1, Table S1. Full baseline characteristics of the analysis cohort by PHPT status; Table S2. Baseline characteristics of the PTH > 65 pg/mL subgroup by PHPT status; Table S3. Missing data before multiple imputation; Table S4. Exhaustive evaluation of predictor combinations; Table S5. Model 2 in the East cohort: incremental and calibration statistics; Table S6. Characteristics of the two subgroups used in the additional analyses.

## Abbreviations

The following abbreviations are used in this manuscript:

25(OH)D	25-hydroxyvitamin D
APH	Autonomous parathyroid hormone hypersecretion
APC	Article processing charge
AUC	Area under the receiver operating characteristic curve
BMD	Bone mineral density
CI	Confidence interval
CRediT	Contributor roles taxonomy
CT	Computed tomography
eGFR	Estimated glomerular filtration rate
EMR	Electronic medical record
IQR	Interquartile range
KDIGO	Kidney Disease: Improving Global Outcomes
MIBI	99mTc-methoxyisobutylisonitrile
MICE	Multiple imputation by chained equations
OR	Odds ratio
PET	Positron emission tomography
PTH	Parathyroid hormone
ROC	Receiver operating characteristic
SD	Standard deviation
SPECT	Single-photon emission computed tomography
TRIPOD+AI	Transparent Reporting of a multivariable prediction model for Individual Prognosis Or Diagnosis + Artificial Intelligence
