# Supplementary material for: A Two-Stage Prediction Strategy for Primary Hyperparathyroidism
Source: Diagnostics (Basel). 2026 Oct 2;16(19):3200. doi: 10.3390/diagnostics16193200 (PMC13663940; doi:10.3390/diagnostics16193200)
Supplement: Supplementary file 1 [file diagnostics-16-03200-s001.zip › diagnostics-4525052-supplementary.pdf]

Supplementary Material

1 Supplementary Tables

Supplementary Table S1. Full baseline characteristics of the analysis cohort by PHPT status.

Variable	Overall (n = 2,788)	Non-PHPT (n = 2,284)	PHPT (n = 504)	P value
**Demographic characteristics**				
Age (years)	59.2 ± 14.3	60.5 ± 14.1	53.4 ± 13.8	<0.001
Male, n (%)	727 (26.1)	582 (25.5)	145 (28.8)	0.143
BMI (kg/m²)	23.5 ± 3.5	23.6 ± 3.6	23.1 ± 3.2	0.033
**Comorbidities**				
Hypertension, n (%)	991 (35.5)	851 (37.3)	140 (27.8)	<0.001
Osteoporosis, n (%)	1171 (42.0)	1027 (45.0)	144 (28.6)	<0.001
Nephrolithiasis, n (%)	323 (11.6)	170 (7.4)	153 (30.4)	<0.001
**Calcium–phosphate metabolism**				
PTH (pg/mL)	43.8 (31.5–61.3)	39.7 (29.4–51.2)	162.0 (99.5–290.2)	<0.001
Total calcium (mmol/L)	2.37 ± 0.28	2.29 ± 0.16	2.76 ± 0.35	<0.001
Albumin-corrected calcium (mmol/L)	2.36 ± 0.26	2.28 ± 0.14	2.71 ± 0.35	<0.001
Phosphate (mmol/L)	1.10 ± 0.25	1.15 ± 0.22	0.84 ± 0.21	<0.001
Magnesium (mmol/L)	0.88 ± 0.10	0.88 ± 0.10	0.88 ± 0.11	0.404
ALP (U/L)	83.0 (66.0–106.2)	79.4 (65.0–99.0)	109.0 (79.0–152.0)	<0.001
Albumin (g/L)	40.9 ± 4.4	40.6 ± 4.4	42.2 ± 4.0	<0.001
**Bone turnover and vitamin D**				
25(OH)D (ng/mL)	16.9 (12.4–22.8)	17.7 (13.0–23.5)	13.8 (9.8–17.6)	<0.001
P1NP (ng/mL)	53.1 (35.4–78.6)	50.7 (34.1–72.5)	80.0 (47.8–136.0)	<0.001
β-CTX (pg/mL)	487.9 (284.0–761.0)	455.5 (271.6–699.2)	699.0 (404.7–1341.0)	<0.001
**Renal function and other indices**				
eGFR (mL/min/1.73 m²)	90.9 ± 22.5	90.4 ± 22.2	93.0 ± 24.1	0.027
Creatinine (μmol/L)	60.7 (51.5–76.4)	60.1 (51.4–75.8)	63.1 (52.1–79.0)	0.032
Urea (mmol/L)	5.6 (4.5–7.0)	5.7 (4.6–7.0)	5.2 (4.2–6.6)	<0.001
Uric acid (μmol/L)	310.0 (255.0–373.0)	304.0 (250.0–367.0)	335.0 (282.0–409.0)	<0.001
Haemoglobin (g/L)	128.4 ± 17.7	128.2 ± 17.5	129.7 ± 18.3	0.043
ALT (U/L)	19.0 (13.0–28.0)	18.0 (13.0–28.0)	20.0 (14.0–30.0)	0.010
AST (U/L)	23.0 (21.0–27.0)	23.0 (21.0–27.0)	24.0 (19.8–30.0)	0.170

Complete version of Table 2, including all measured variables. Values are mean ± standard deviation, median (interquartile range) for skewed variables, or n (%). Bone turnover markers (P1NP, β-CTX) differed markedly between groups but were not used as predictors because of substantial differential missingness (Supplementary Table 3). Abbreviations as in Table 1.

Supplementary Table S2. Baseline characteristics of the PTH > 65 pg/mL subgroup by PHPT status.

Variable	Overall (n = 623)	Non-PHPT (n = 154)	PHPT (n = 469)	P value
**Demographic characteristics**				
Age (years)	53.8 ± 14.3	56.3 ± 15.8	52.9 ± 13.6	0.025
Male, n (%)	188 (30.2)	49 (31.8)	139 (29.6)	0.682
BMI (kg/m²)	23.4 ± 3.3	23.9 ± 3.5	23.1 ± 3.2	0.024
**Comorbidities**				
Hypertension, n (%)	192 (30.8)	64 (41.6)	128 (27.3)	0.001
Osteoporosis, n (%)	170 (27.3)	42 (27.3)	128 (27.3)	1.000
Nephrolithiasis, n (%)	183 (29.4)	30 (19.5)	153 (32.6)	0.003
**Calcium–phosphate metabolism and vitamin D**				
PTH (pg/mL)	141.0 (93.2–253.5)	91.8 (75.8–115.0)	169.0 (109.0–304.0)	<0.001
Total calcium (mmol/L)	2.67 ± 0.38	2.32 ± 0.26	2.79 ± 0.34	<0.001
Albumin-corrected calcium (mmol/L)	2.64 ± 0.37	2.30 ± 0.25	2.74 ± 0.34	<0.001
Phosphate (mmol/L)	0.87 ± 0.23	1.04 ± 0.22	0.82 ± 0.20	<0.001
ALP (U/L)	101.0 (75.1–140.0)	81.0 (64.0–103.0)	111.0 (80.8–156.0)	<0.001
Albumin (g/L)	42.0 ± 4.0	41.0 ± 3.8	42.2 ± 4.0	<0.001
25(OH)D (ng/mL)	13.8 (10.1–18.2)	15.2 (12.4–20.8)	13.4 (9.6–17.2)	<0.001
**Renal function**				
eGFR (mL/min/1.73 m²)	93.0 ± 24.1	92.1 ± 23.3	93.2 ± 24.3	0.389

The population in which Model 2 was developed and validated. Only 35 participants with PHPT had a PTH of 65 pg/mL or below, none of whom had nephrolithiasis; counts for that comorbidity are therefore identical to those in Table 2. Abbreviations as in Table 1.

Supplementary Table S3. Missing data before multiple imputation.

Variable	Missing (n)	Missing overall (%)	Missing in non-PHPT (%)	Missing in PHPT (%)
Total calcium (mmol/L)	0	0.0	0.0	0.0
Albumin-corrected calcium (mmol/L)	69	2.5	2.8	1.0
ALP (U/L)	5	0.2	0.2	0.2
Age (years)	0	0.0	0.0	0.0
PTH (pg/mL)	0	0.0	0.0	0.0
Phosphate (mmol/L)	2	0.1	0.0	0.2
Albumin (g/L)	69	2.5	2.8	1.0
Magnesium (mmol/L)	10	0.4	0.3	0.8
Haemoglobin (g/L)	17	0.6	0.3	2.0
Uric acid (µmol/L)	3	0.1	0.1	0.0
Urea (mmol/L)	0	0.0	0.0	0.0
eGFR (mL/min/1.73 m²)	0	0.0	0.0	0.0
ALT (U/L)	7	0.3	0.2	0.4
25(OH)D (ng/mL)	119	4.3	0.6	21.0
P1NP (ng/mL)	132	4.7	0.7	22.8
β-CTX (pg/mL)	132	4.7	0.7	22.8
BMI (kg/m²)	182	6.5	1.5	29.4

Missingness before multiple imputation, overall and by outcome status. Missingness was negligible for all variables entering either model but was substantially higher among participants with PHPT for BMI and the bone turnover markers, none of which were used as predictors. Multiple imputation by chained equations ($m = 10$, Bayesian ridge regression) was performed with the outcome and 17 auxiliary variables in the imputation model. Abbreviations as in Table 1.

Supplementary Table S4. Exhaustive evaluation of predictor combinations.

Model	Combinations evaluated	Rank of final model (mean external AUC)	Difference from highest-ranking combination	Top-20 overlap between East and Caotang rankings
Model 1	1392	above 90th percentile	0.007	1 of 20
Model 2	682	above 90th percentile	0.009	2 of 20

Predictors were selected on pathophysiological grounds and on availability in routine practice rather than by an automated procedure. Both models were finalised in the development cohort before this combination analysis was carried out; the analysis is post hoc and exploratory, and the external cohorts contributed nothing to predictor selection. To quantify the cost of not optimising variable selection, all combinations of up to four candidate predictors were evaluated within the availability constraints applying to each model. Both final models ranked above the 90th percentile of mean external AUC. Rankings were, however, not reproducible between the two external cohorts. With 98 and 73 outcome events in those cohorts, this lack of reproducibility indicates imprecision and insufficient power rather than robustness of the selected models. Serum phosphate was excluded from the Model 1 candidate pool on grounds of availability rather than performance. When phosphate was admitted to that pool, calcium + phosphate + ln(alkaline phosphatase) ranked 69th of 1,391 combinations (upper 5.0%; mean external area under the curve 0.886) against 137th (upper 9.8%; 0.868) for Model 1, and all twenty highest-ranking combinations contained phosphate.

Supplementary Table S5. Model 2 in the East cohort: incremental and calibration statistics.

Statistic	Value
Participants (events / non-events)	105 (91 / 14)
Model 2 AUC (95% CI)	0.870 (0.778–0.963)
PTH alone AUC	0.811
ΔAUC	+0.060
P value (DeLong)	0.231
Calibration slope	0.75
Calibration-in-the-large	1.01

The Model 2 subgroup of the East cohort contains only 14 outcome-negative participants, far below the number required for reliable estimation of incremental discrimination or of calibration. These statistics are therefore reported here rather than in the main text and should not be read as evidence either for or against incremental value in that cohort. The pooled East and Caotang estimate (Table 4) is presented as the primary external result for Model 2. Abbreviations as in Table 1.

Supplementary Table S6. Characteristics of the two subgroups used in the additional analyses.

Variable	PTH >65 pg/mL and calcium within reference range (n = 214)	Positive histopathology (n = 88)	PTH normalised after vitamin D repletion, calcium within reference range (n = 131)
PHPT, n (%)	83 (38.8)	88 (100)	0 (0)
Age, years, median (IQR)	55 (47–65)	52 (45–58)	56 (47–67)
Female, %	70.1	69.3	70.2
PTH, pg/mL, median (IQR)	98.6 (77.8–131.2)	203.5 (128.2–374.2)	87.7 (75.3–108.0)
Total calcium, mmol/L, median (IQR)	2.28 (2.18–2.41)	2.75 (2.55–2.95)	2.25 (2.16–2.31)
Phosphate, mmol/L, median (IQR)	1.00 (0.85–1.14)	0.82 (0.68–0.94)	1.07 (0.93–1.19)
ALP, U/L, median (IQR)	82.0 (67.0–109.0)	118.0 (87.8–166.0)	79.0 (63.8–97.0)
25(OH)D, ng/mL, median (IQR)	14.6 (11.8–20.7)	14.9 (9.3–20.0)	14.7 (12.2–21.2)
eGFR, mL/min/1.73 m^2, median	98.9	102.8	97.3
Osteoporosis, %	22.9	23.9	27.5
Nephrolithiasis, %	18.2	36.4	16.0

The subgroup on the left is the population used for the analysis of Model 2 in participants with elevated PTH and a calcium within the reference range (S4). The two right-hand columns are the case and comparison groups of the focused analysis (S5), in which the reference standard rests on histopathology and on the response to vitamin D repletion respectively. Both are highly selected and are not representative of the full analysis cohort. Abbreviations as in Table 1.
